# A cross-case comparative analysis of international security forces’ impacts on health systems in conflict-affected and fragile states

**DOI:** 10.1186/s13031-015-0040-y

**Published:** 2015-04-13

**Authors:** Margaret Bourdeaux, Vanessa Kerry, Christian Haggenmiller, Karlheinz Nickel

**Affiliations:** Division of Global Health Equity, Brigham and Women’s Hospital, 75 Francis Street, Boston, MA USA; Harvard Medical School, Department of Global Health and Social Medicine, 641 Huntington Avenue, Boston, MA 02115 USA; Avenida Tenente Martins, Monsanto, 1500-589, Lisboa, Portugal; Independent Research & Consultancy, Rua de Angola 92, 2765-193 Estoril, Portugal; Associate Director of Partnerships and Global Initiatives, Center for Global Health, Massachusetts General Hospital, 100 Cambridge Street, 15th floor, Boston, MA 02114 USA

**Keywords:** Health systems, Fragile states, Fragile situations, Civil-military interaction, Security forces, Health system reconstruction, Stabilization, Health system strengthening

## Abstract

**Background:**

Destruction of health systems in fragile and conflict-affected states increases civilian mortality. Despite the size, scope, scale and political influence of international security forces intervening in fragile states, little attention has been paid to array of ways they may impact health systems beyond their effects on short-term humanitarian health aid delivery.

**Methods:**

Using case studies we published on international security forces’ impacts on health systems in Haiti, Kosovo, Afghanistan and Libya, we conducted a comparative analysis that examined three questions: What aspects, or building blocks, of health systems did security forces impact across the cases and what was the nature of these impacts? What forums or mechanisms did international security forces use to interact with health system actors? What policies facilitated or hindered security forces from supporting health systems?

**Results:**

We found international security forces impacted health system governance, information systems and indigenous health delivery organizations. Positive impacts included bolstering the authority, transparency and capability of health system leadership. Negative impacts included undermining the impartial nature of indigenous health institutions by using health projects to achieve security objectives. Interactions between security and health actors were primarily ad hoc, often to the detriment of health system support efforts. When international security forces were engaged in health system support activities, the most helpful communication and consultative mechanisms to manage their involvement were ones that could address a wide array of problems, were nimble enough to accommodate rapidly changing circumstances, leveraged the power of personal relationships, and were able to address the tensions that arose between security and health system supporting strategies. Policy barriers to international security organizations participating in health system support included lack of mandate, conflicts between security strategies and health system preservation, and lack of interoperability between security and indigenous health organizations with respect to logistics and sharing information.

**Conclusions:**

The cases demonstrate both the opportunities and risks of international security organizations involvement in health sector protection, recovery and reconstruction. We discuss two potential approaches to engaging these organizations in health system support that may increase the chances of realizing these opportunities while mitigating risks.

## Background

Armed conflict in fragile states erodes health systems, where health systems are defined as the organized network of institutions, resources and people that deliver health care to populations. Research shows destruction of these health systems, or sectors, is a primary reason for persistently high mortality and morbidity in conflict-affected and fragile states (CAFS) for years after hostilities cease [1]. In these absences of robust health systems, international health initiatives also fail to achieve their goals since programs cannot be effectively implemented [2]. The majority of Millennium Development Goals will go unmet in the 35 fragile countries listed by the World Bank [3].

What can be done to protect and quickly recover health systems in fragile and conflict-affected states? There has been significant debate in the past decade both about conceptualizing and how best to support elements of health systems in CAFS. In 2004, the World Health Organization offered a description of health systems based on six ‘building blocks’, or inputs. These included governance, finance, health services, information systems, medicines and technologies, and workforce. For outcomes, there is loose consensus that functioning health systems should improve the health of the population, achieve high levels of public satisfaction with services, and protect citizens from social and financial risk [4,5].

International efforts to support health systems in CAFS are directed toward a variety of these building blocks and outcomes. Initiatives have ranged from creating new health governance and performance assessment strategies [6,7] to strengthening health finance mechanisms [8], resurrecting health workforces, and repairing and reconfiguring health delivery services [9].

However, a growing body of experience and research notes that groups outside of the health sector significantly shape the political, economic, and security environment in which health sector support takes place [10]. Health system researchers Frenk and Moon [11] note that health sector actors, both domestic and international, often undertake health system interventions isolated from key non-health actors. Writing with respect to global health priorities generally, they remark, “Global health is increasingly the product of cross-sector interdependence—that is, the outcome of policymaking processes across multiple sectors. However, global health actors today are largely unequipped to ensure that health concerns are adequately taken into account in crucial policymaking arenas such as trade, investment, security, the environment, migration, and education.”

These issues are particularly acute in fragile state settings where diverse group of international health actors undertake health system interventions in volatile political and economic circumstances. Health system analysts Colombo and Pavignani, of the World Health Organization, underline, “In the political deals between governments, rebels, UN agencies, donors, development banks, private companies and providers, foreign armies, and peacekeepers, important decisions that affect the health sector and shape the decision space of its actors are taken…yet [health sector] policy discussion is often kept within a narrow circle of health professionals who may be remarkably unaware of the influence of political, economic, legal, and administrative determinants on health developments.” [10].

One particular group of non-health actors plays a particularly significant role in fragile states: International security forces (ISFs). ISFs deployed to fragile states with a mandate to protect civilians and promote stability are key actors relevant to shaping the environment in which health sector interventions take place. Yet, to date there is limited systematic work examining the scope of impacts of international security organizations on health sectors. Their broad mandates, vast resources, and breadth of engagement in fragile states, however, raise the possibility they do impact health systems in, as of yet, undocumented ways. This constitutes a problem for actors focused on the health system: with little insight into the ways ISFs might impact health system supporting efforts, they will remain unable to anticipate the negative impacts ISFs might have on the process of health system protection, recovery and reconstruction; conversely they will remain unable to leverage the resources and assets ISFs might lend to health system support, or evaluate the risks inherent in utilizing these resources.

### International security forces and health

Discussions about the role of ISFs in health largely focus on the robust debates about ISFs directly providing short term health services in order to achieve a tactical military objective, like currying the favor of a particular person or group of people. Concerns about these ‘quick impact projects’ predominantly regard their subversion of the impartiality of humanitarian health services [12,13]. A number of important guidelines have been developed that aim to constrain these activities and reserve the involvement of militaries in relief efforts for exceptional cases, including the “The Use of Foreign Military and Civil Defense Assets in Disaster Relief (the Oslo Guidelines)” and “Civil-Military Guidelines during Complex Emergencies” [14,15]. Likewise guidelines relevant to military-humanitarian relations and forums to guide the interactions between militaries and humanitarian organizations have also evolved. For example, the UN Office for the Coordination of Humanitarian Affairs (OCHA) manages some of these interactions, providing training for UN peacekeepers and hosting round tables and conferences specific to regions where these organizations may come into conflict with one another [16]. The UN health cluster system creates an information-sharing forum for the plethora of health organizations providing humanitarian health aid during emergencies and has issued guidelines with respect to working with militaries to delivery humanitarian health aid [17]. Further, various ISFs and health NGOs have variations of ‘civil-military’ liaisons with a diversity of aims and goals [18]. These forums and mechanisms represent a huge step forward in clarifying roles and relationships when addressing emergency humanitarian health needs of people in fragile settings. However, because they are framed in terms of humanitarian health aid delivery, their application to issues of health sector protection, recovery and reconstruction is limited. Several papers including the Humanitarian Policy Group at the Overseas Development Institute, Trends and challenges in humanitarian civil-military coordination” [18] and the Inter-Agency Standing Committee’s Civil-Military Relationship in Complex-Emergencies” provide in-depth reviews of these debates [19].

However, while important and related, these discussions of ISFs’ role in providing short-term humanitarian health care services and their relationship to humanitarian organizations are insufficient. The size, scope, scale and political influence of many ISFs suggests their impact on health systems run deeper than just the effects on short-term humanitarian health aid. There is an array of ways ISFs may influence health systems overall which need to be understood.

Second, an exclusive focus on military involvement in humanitarian aid delivery signals that humanitarian aid is the only health related issue relevant to the security community in CAFS. Since ISFs can never, by definition, be humanitarian actors on account of their lack of neutrality and independence from governments, their role in humanitarian aid delivery should be limited if not absent. As a consequence, security organizations receive the message that there is very little need to consider how they impact health systems beyond adhering to the Geneva Conventions’ rules on avoidance of targeting health infrastructure and personnel and honoring guidelines limiting their participation in humanitarian aid delivery [20].

The purpose of this paper, then, is to describe the breadth of impacts ISFs have on health systems in the complex environment of CAFS and to explore, in instances where ISFs did impact health systems, the forums, mechanisms, and policies that influenced their interactions with health system actors. To achieve this, the paper provides a cross case comparative analysis of four cases in a diverse set of fragile state contexts to document and analyze:

- the array of instances when ISFs impacted one or more of the six WHO building blocks of fragile states’ health systems
- the forums, or mechanisms by which ISFs interact with health sector actors during this process, and
- the policies that facilitate or hinder ISF support of health system building blocks in CAFS.

## Methods

Our goal was to identify the common patterns by which ISFs impact health sector building blocks, engage with health sector actors, and identify policies that constrain or facilitate their engagement so that health sector actors can predict how ISFs may behave in future fragile state contexts. One method by which to generate predictive findings is by using a comparative method called the ‘most different’ approach [21] or ‘the method of agreement’ [22]. Here the strategy is to compare cases where all major elements differ except for the phenomena in question. The power of this approach lies in forcing researchers to confront “such a broad range of cases that they must distill out of that diversity a set of common elements that prove to have great explanatory power” [23].

We chose to compare cases that differed, notably by geography, reason for international intervention, type and size of ISF, and timeframe of ISF involvement, so we could see how ISFs consistently impacted health sectors, engaged with health sector actors, and were constrained by policies, despite these variations. We describe how we conducted the individual cases below and follow it with a description of the methods for the comparative analysis.

### Conducting the case studies

We conducted a series of four case studies in Haiti [24], Kosovo [25], Afghanistan [26] and Libya [27]. The purpose of these cases was to identify and describe in detail major examples of ISF interactions with health systems in fragile state contexts, the forums or mechanisms by which they interacted with health system actors, and the policies that facilitated or hindered these interactions, as perceived by participants in the case studies.

The cases were conducted, analyzed and published between September 2011-December 2013. Three criteria were established for case country selection. First, all case countries were defined as fragile situations by the World Bank [3]. Second, in each case a human security crisis, such as natural disaster, ethnic conflict, intra- or inter-state conflict, or insurgency, threatened the health system and further, there was a global crisis response directed towards supporting the health system. Third, a multinational security force with a peace keeping, peace building, or stabilization mandate was present. We chose the resulting cases to represent a broad range of circumstances so findings would be applicable to a variety of contexts.

We defined ‘international security force’ as an institution that carried an internationally sanctioned mandate to use force to restore public order and maintain or enforce peace. This included militaries, military coalitions, police, intelligence agencies, peacekeeping, and peace enforcement missions and encompassed their political leadership bodies. It also included ‘rule of law’ missions, such as those conducted by the European Union, where the mission has the power to investigate, arrest, prosecute and imprison citizens of a fragile state. Of note, we did not interview indigenous security forces or non-state armed groups. These actors undoubtedly impact health systems, but their interactions with the health sector and the policy prescriptions that may change their behavior were beyond the scope of the case studies.

We chose this expansive definition of ISF for two reasons, one practical and one aspirational. Practically ISF composition in fragile settings varies across cases. For example, in some cases foreign militaries run anti-corruption initiatives or take on a policing role. In other cases foreign civilian organizations play these roles [28]. We wanted our findings to apply to this wide array of organizations involved in the security sector that may possess different titles but fulfill similar functions.

Secondly, a large number of both health and security organizations and the fractured arrays of lines of leadership, guidance messages, mandates, goals and strategies undermine efforts at maintaining focus on a common goal of supporting indigenous institutions in fragile states. In our attempt to generate findings applicable to all international organizations involved in the security sector, we hope to lend coherence and consistency to the process of health system support on the part of security actors. This speaks to our aspiration for security organizations and their political leadership to internalize the World Health Organization’s message that health systems are indeed ‘everybody’s business’ [4].

For the purposes of devising an investigative plan, we used the six World Health Organization (WHO) building blocks of health systems as a template for investigation, exploring how each building block was affected by a crisis in each case. We examined three distinct time points of the crisis including: a) immediately prior to ISF intervention, b) during intervention, c) and, if applicable, after intervention until the present day.

We first conducted literature reviews and background interviews with respect to each building block of the country’s health system and with respect to the international security forces present. We reviewed scholarly articles, public reports, organization documents, lecture and newspaper articles about each country’s health system and how crisis affected it. We also reviewed organizational documents pertinent to guidelines governing civilian-military interaction, mandates of security organizations with respect to delivery of health care, protecting instruments of government and civilian protection.

We identified key informants during this process including indigenous health sector leadership, indigenous civilian emergency workers, health NGO leaders, donors engaged in health system support, military members acting as liaisons with civilian health groups, and leaders of UN, NATO, or and individual country’s security mission. We also used these reviews to triangulate information later gleaned from interviews.

Next, we conducted semi-structured interviews. Key informants were invited to participate via email. We obtained verbal informed consent before commencing interviews, including an explanation that information gleaned from interviews was not for attribution. Interviews were semi-structured around the themes of the WHO building blocks and tailored to the interviewees’ background and known involvement in the health sector. Each interview probed for instances of interactions between ISFs and health system building blocks.

Other key informants were identified through the process of “snow ball” interviewing whereby interviewees identify others who are relevant to the subject at hand. Those people were in turn invited to interview, with the process continuing until the point of repetition when only informants who had already been identified were mentioned.

These interviews were conducted in the field in both Haiti and Kosovo. Because of security concerns, fieldwork was not possible in Afghanistan and Libya, although several primary research team members had deployed to Afghanistan multiple times. Every effort was made to interview key informants in person when they traveled outside of the country. When this was not possible, interviews were conducted by phone.

During these interviews, we flagged any mention of ISF involvement in any of the health system, and coded them by building block. After reviewing the interview transcripts, the research team discussed and mutually agreed upon what the major examples of ISF engagement or impact on the health system were in each case. We then generated an investigative plan to explore these examples, with the goal of writing a ‘narrative’ detailing the example in-depth.

The investigative plans for each narrative included again literature reviews and key informant interviews. Two sets of questions were the focus of each narrative investigation. The first set addressed the type and nature of ISF involvement in the health sector: what building block(s) did the ISF impact? In the view of participants, was this involvement helpful, harmful, mixed, or irrelevant? In the participants view, were there ‘missed opportunities’ when ISFs did not deploy capabilities that could be valuable to supporting health systems? The second addressed interactions with health sector actors and policies that influenced those interactions: What were the forums and mechanisms by which security actors interacted with health sector actors, if any? Were they formal or informal? How were roles negotiated? Did they rely on any policy guidance when planning their interventions? How did these interventions relate to the ISF’s organization mandate and internal policies? In the interviewee’s view, did these mandates and policies help to define the ISF’s role and did they facilitate positive ISF impacts on the health system?

We generated case reports by first summarizing the health system of the case country, the international intervention in the country’s health system, and the types and mandates of international security actors in the case. The narratives, 2-3 per case, comprised the second half of the case study reports.

### Conducting the cross case analysis

We carried out the cross-case analysis by reading and re-reading the four compiled case studies, eleven included narratives, and the original case study interview transcripts [29]. The analytic process was similar to the individual case analysis in that data from these sources were again grouped according to:

- How and which building blocks of the health system did ISFs repeatedly engage or impact? In the view of participants what was the effect of this impact on health system outcomes?
- What forums or mechanisms did ISFs utilize to interact with health sector actors?
- What were the policy issues that facilitated or hindered ISF contributions to health system protection, recovery or reconstruction?

Common themes with respect to these questions were identified and discussed among the team. Team consensus about the answers to the above questions was achieved through repeated discussion and reviewing of the primary data.

## Results

We first summarize the four case studies and then, drawing on examples from the cases, answer the three primary questions of the cross case analysis.

### Case summaries

Haiti: The Haitian case study examined the role of ISFs, including the US military, UN Peacekeepers, and other bilateral foreign militaries engaged with Haiti’s health system after the 2010 earthquake. The case contained three narratives. The first examined the US military’s involvement in reestablishing the health system’s medical supply networks and health infrastructure. How this impacted the authority and legitimacy of indigenous health sector leaders was examined. The second explored the use of bilateral militaries deployment of tertiary care facilities. While these facilities were not aimed at health system support, the narrative considered how such resources could bolster indigenous health service delivery organization capacity in similar scenarios. The third narrative discussed how cholera, introduced to Haiti accidentally by UN peacekeeping forces, impacted the fragile health system. We discussed how such instances might be prevented in future operations and explored how security organizations possessing surveillance, engineering, and logistics resources, could potentially mitigate the impact of similar public health threats [24].

Coordination mechanisms to incorporate security organizations’ contributions relied heavily on personal relationships. Formal coordination forums did emerge but only after the establishment of trust between individual security and health actors. When security and health system actors did tackle a joint problem, the lack of interoperability in communication and logistics systems reduced their effectiveness. Overarching policy issues included the mandates of security organizations, which focused on providing emergency relief but not protecting and bolstering indigenous health, food and water systems [24].

Kosovo: Kosovo’s case focused on NATO’s intervention in Kosovo during the 1999 war through the present day, and the civilian European Rule of Law mission, EULEX, which strives to reduce high-level corruption in Kosovo’s post-war government. The first narrative discussed strategies for international rule of law missions to prevent health sector corruption from taking root in similar missions. The second narrative looked at the need for robust epidemiologic surveillance systems in fragile states and examined how NATO’s new epidemiologic surveillance system that detects epidemics among troops might also feed into the World Health Organizations’ emerging disease surveillance system, EWARN. The third narrative told the story of NATO’s response to its discovery of lead contamination in Northern Kosovo. The health, economic, and political opportunities and challenges involved in NATO detecting this public health threat were explored [25].

Coordination strategies between health and security actors remain largely ad hoc and underutilized according to study participants. While many health actors were open to engaging the security community in health system support issues, it was unclear who to speak with or how to work with these organizations. Policy issues again included the mandates and scope of work of security organizations as well as the low political value placed on health system improvement and reform [25].

Afghanistan: The Afghanistan case examined the role of the US military, NATO forces, and the UN peacekeeping mission in Afghanistan’s newly minted health sector. One narrative explored the scale and scope of NATO’s program to create a health system for Afghanistan’s military and security personnel and discussed how this effort impacted the health system overall. Foreign militaries impact on the Afghan Ministry of Health’s participation in the Global Polio Eradication Campaign served as the focus of the second narrative. The narrative detailed how NATO coalition forces arrived at a ‘passive support’ strategy of the polio campaign in an effort to lessen their political impact on the Ministry of Health. The third and final narrative examined ISFs’ negative impacts on health delivery and governance through health-related counterinsurgency projects. It examined the tensions between, as well as the opportunities to reconcile the goals of counterinsurgency and health system strengthening [26].

A plethora of coordinating bodies and mechanisms arose over the past decade of conflict in Afghanistan. Interestingly, most of them took years to materialize. The issues covered in the narratives were rarely informed by early coordination with health sector actors. Those that were utilized were often stymied by high staff turnover on the part of security and health organization personnel. The policy issues that loomed large in Afghanistan where ISFs’ use of highly controversial health ‘hearts and minds’ operations sowed distrust and anger on the part of health sector actors who saw this strategy as undermining the impartiality of indigenous health care institutions and turning seeking of health care services into a political act on the part of civilians [26].

Libya: The Libya Case focused on NATO’s civilian protection mission in Libya in 2012. Two issues were explored in the case body and through two narratives. The first was NATO and OCHA’s use of new strategies for protecting health system infrastructure and workforce. The narrative investigated how ISFs can best gain awareness of health system threats and communicate with health system actors using these new approaches. The second narrative focused on Libya’s program to care for its war wounded by sending them abroad for care. The program, riddled with fraud, drained the Ministry of Health budget and weakened the new government of Libya. The narrative noted how ISFs can potentially play a helpful role in supporting essential care for the war wounded in fragile states and thus improve the capacity and functioning of indigenous health service organizations [27].

Coordination issues were the focus of the first narrative regarding how new forums and information platforms could be utilized to improve situational awareness of the health sector. Policy issues included NATO’s lack of mandate to participate in institution recovery or rebuilding after the cessation of hostilities, even though they may have improved health sector reconstruction in retrospect [27].

### What health system building blocks were impacted by ISFs in the cases?

ISF impacts’ on the cases’ health systems fall into three ‘building block’ groups: health sector governance, health information systems and indigenous health services.

### Health sector governance

ISFs impacted health system governance in two respects. First ISFs created parallel health systems or sub-systems that circumscribed the resources and power of the state’s health system leadership. Second, they performed regulatory functions for health systems.

Foreign militaries established parallel health systems or sub-systems in Afghanistan, Kosovo and Haiti. This process was most extensive in Afghanistan, where donor government militaries invested heavily in a completely separate health system for Afghanistan’s military and police [26]. The impetus was in response to the attrition rate of the Afghan forces from illness which was more than 18 times than from battlefield injury [30]; thus efforts to build an Afghan security force hinged on improving the health of personnel. In response, ISFs created dozens of health facilities and developed medical training programs with the goal of providing healthcare to over 1 million beneficiaries. As a result, different government departments—the Ministry of Health and the Ministry of Defense—housed the civilian and military health systems, both competing for donor funds and health workers [26]. Likewise, in Kosovo and Haiti, foreign militaries financed the health systems’ disaster and public health emergency response capabilities [25,31,32]. These programs also lived outside the Ministries of Health, and according to case study participants, required the health ministries to have to negotiate with other government officials to leverage necessary resources to respond to public health threats. In Haiti in particular, the practice of housing assets outside the Ministry of Health’s control rendered it unable to respond to public health emergencies [33]. Moreover, since security organizations harbored considerable logistics capabilities—transport, communications, and security—sometimes health officials negotiated directly with security actors to acquire the assets necessary to manage a public health crisis [24].

In the cases, ISFs also were involved in regulatory functions of the health system. At times, they enforced the laws of the health system and/or bolstered the authority and accountability of its leadership. Notably, ISFs fielded anti-corruption campaigns in both Kosovo and Afghanistan [25,26]. These initiatives addressed instances of widespread health system procurement fraud and involved the arrest of high-level ministry officials [30,34]. In Afghanistan, Haiti and Kosovo, ISFs bolstered and extended the authority of health leadership. For example, in Haiti, health officials requested US military personnel to confront foreign aid workers not following the ministry’s policies [24]. In Afghanistan, when coalition forces initially provided health care to civilians, evidence mounted that this undermined the competence and capability of the Ministry of Health in the eyes of some populations, who perceived their own government to be unable to delivery essential services. Because of these findings, coalition forces developed alternative programs to implement the Ministry of Health’s national health agenda in remote and insecure districts with the hope it reversed these unintended consequences [35]. In Kosovo, health officials worked to gain the backing of ISF leadership so as to advance the Ministry’s health reform laws. One Kosovar ministry official explained that security forces carried enough authority to be able to put health issues on the government’s agenda. “If only one representative from NATO would come with me to our intergovernmental meetings to put [health reform] on the national agenda that would be enough to make progress [in advancing the health reform law]” [25].

### Health information systems

The WHO guide “Analyzing Disrupted Health Sectors” notes, “the strongest indicator of crisis is lack of data” [10]. This truth is evident throughout each of the four cases. Baseline assessments of health system performance prior to the crisis were absent or inaccessible to both health system leadership and international crisis responders. Even information like the location of basic health infrastructure was absent in some instances [36]. Situational awareness, or continuous assessment monitoring during a crisis period, was limited because of lack of collective forums to report, find or collate real-time information. Post-crisis health threat surveillance systems did not link to appropriate action plans for detected threats, leading to diffusion of responsibility across response organizations and fragile state governments. For example in one instance in Kosovo, NATO forces alerted UN authorities in 2000 to lead contamination in the Mitrovica region, particularly affecting residents of an internally displaced persons camp in the area. Because it was unclear whose responsibility it was to act on such information, it was not until 2005—five years later—that international organizations undertook efforts to relocate camp residents [25].

These health information problems hindered security organizations’ assistance to health sectors. In Haiti, lack of basic information about the health sector, such as maps of the country or where clinics and hospitals were located, meant military earthquake responders initially did not know where to send medical resources [24]. Likewise, in Kosovo the chaos of the post-conflict period apparently obscured looting of medical institutions and targeting of health workers until too late for policy makers to direct security forces to protect them [37].

We found examples throughout the cases of when security organizations both contributed to or missed the opportunity to contribute to health information shortfalls or uptake health sector information when it was available. In Haiti, militaries contributed valuable drone and satellite imagery of the earthquake-affected area that civilian volunteers then used in their remarkable effort to generate detailed maps used by all crisis responders, including militaries [38]. In Kosovo, NATO developed a sophisticated epidemiologic surveillance system that could rapidly detect outbreaks of communicable diseases. This surveillance tool currently is focused exclusively on detecting disease outbreaks among deployed soldiers. If policy barriers regarding sharing of information between security and health actors could be overcome, this epidemiologic surveillance system could be leveraged to detect public health threats to the civilian population as well [25]. In Libya, OCHA worked with volunteer ‘crisis mappers’ to document population movements and the functional status of health facilities. However, although this information may have been useful to militaries in avoiding harm to people and facilities, they were reluctant to use it because it came from novel and internally unverified sources and methods [27].

In Haiti, the UN mission established a sophisticated information gathering operation to detect early signs of civil unrest, monitor crime, and political instability. Public health threats and epidemics, which do inflame local tensions and can stoke civil unrest, were not included in this surveillance operation. However, monitoring critical health data through these operations could help sensitize UN Heads of Mission to the political and societal impacts of public health emergencies, enabling them to better allocate resources to addressing them [24].

### Indigenous health service delivery

Indigenous health delivery service organizations—those run or managed through the country’s health sector—were heavily damaged or completely destroyed in every case. Of note, we drew a distinction between ISF’s influence on humanitarian health aid delivered by humanitarian NGOs and ISF influence on indigenous health care organizations native to the country’s health sector. In some cases the lines between these two were blurred as when a particular foreign NGO had been present in a state for so long it might be viewed as native to the health sector. Further, concerns about an ISF’s impact on the impartiality of the health sector paralleled similar concerns about ISFs and their impact on humanitarian neutrality, impartiality and independence. Nevertheless, for the purposes of this project we maintained this distinction for conceptual clarity.

Case analysis showed that security organizations had significant impacts on indigenous health care services in four ways.

First, because they are armed, security organizations possess the capability to directly protect or destroy health system service programs and their necessary assets, like buildings and supplies. In Libya, there was an explicit effort by NATO to identify and protect health service organizations and programs. As a result, NATO managed to avoid causing direct damage to indigenous health organization infrastructure [27]. In Haiti, militaries guarded health care organization infrastructure and helped reestablish medical distribution points for indigenous health care organizations [39]. In Kosovo, however, NATO and UN peacekeepers were unable to protect Serbian health workers. Seeking refuge in protected enclaves, the vast majority left the Kosovar health system. This contributed to the creation of a parallel health system for Serbian citizens, which persists to this day. Later, however, NATO forces became instrumental in protecting patients and medical supply routes in this parallel system [37,40]. While we did not explicitly investigate nor uncover instances of security forces directly targeting health system assets, the International Red Cross/Red Crescent Society report on Health Care in Danger does document dozens of instances when they have: security forces can pose a direct threat [41].

Of note, protection of health service programs may require intentional distance from security actors. In Afghanistan, the visible proximity of NATO troops to the Afghan Ministry of Health’s participation in the Global Polio Eradication Campaign (GPEC) was a significant problem. The population assumed GPEC was a formal military run health program and it became a target for Taliban-aligned forces [26]. After extensive negotiations with the Ministry of Health, NATO adopted a “passive support” policy whereby NATO forces would remain as far away as possible from the campaign while engaged in ceasing hostilities nearby [42].

Second, in every case, militaries and security groups provided direct medical care to civilians. Empowered by the medical resources necessary to care for their personnel, militaries possessed high value medical assets. These resources were allocated to civilians as well but their relative impact on indigenous health service delivery varied widely across each country. In one important example in Afghanistan, NATO medical forces used medical projects to generate good will toward the coalition forces and to collect human intelligence [43]. This practice had many negative impacts. It was widely condemned by Afghan officials and international aid workers alike and compromised the impartiality of healthcare [44]. It also undermined the authority of the Afghan Ministry of Public Health by signaling to civilians that health care provision was the domain of foreign militaries and not of their own government. An internal investigation by NATO confirmed the approach’s inefficacy and divisiveness, and coalition forces eventually transformed their medical programs to be owned and run by the Afghan Ministry of Public Health [43].

In Haiti and Libya the role of direct care delivery on the part of militaries could have bolstered the capacities of indigenous health service organizations according to case study participants. Twenty-six militaries provided health care in Haiti, with three—the US, Canadian, and Israeli—providing tertiary care services [24,45]. The US and Canadian militaries also provided helicopter patient transport services. Although no quantitative data was available from the case to confirm, health providers argued these services increased indigenous health organizations’ capacity by unburdening them of the sickest, most resource intensive patients [46]. Similarly in Libya, the newly minted Ministry of Health for political reasons was forced to continue a three billion dollar, corrupt, and unsustainable program to send citizens wounded in the war to other countries for care. Ministry officials postulated they would have had a much stronger political negotiating position to end the program had ISFs offered short-term trauma care for war wounded as an alternative [27].

Third, security organizations’ logistics capabilities were leveraged to assist indigenous health service organizations resume provision of health services. The US military in Haiti provided the most significant example of this; they fielded 10,000 soldiers who reopened supply routes into the country. These forces were able to open the single strip airport in Port au Prince within hours of the earthquake and cleared rubble from and organized the medical warehouse that served as the main distribution point for medications [47].

Finally and fourth, security organizations directly impacted the population’s burden of disease and the subsequent burden on the sectors’ health service programs. The most significant example again was in Haiti, where security actors inadvertently contaminated the country’s water supply with cholera; the cholera became epidemic killing thousands, sickening hundreds of thousands and remains now endemic in the country [48].

### Forums and mechanisms for ISF-health system actor interaction

Purposeful planning to support health systems was the exception rather than the rule in the four cases. Few, if any, standing formal institutional coordination mechanisms existed between security organizations and international or state health agencies. Rather, coordination mechanisms and policies had to be negotiated in the middle of crises and often in reaction to complaints in each instance. As mentioned in Afghanistan, prolonged negotiations with Ministry of Health officials finally led coalition forces to reform their policies for direct care provision and support for the Global Polio eradication campaign [26]. In Haiti mounting public complaints from health NGOs compelled the US military forces and US State Department to respond and invent a structured system for prioritization of medical and relief supplies in the supply chains they were tasked with reconstituting and managing [49].

Helpful support from security organizations was facilitated by personal relationships between security and health system actors. Both mundane tasks such as negotiating patient transport mechanisms and large-scale responses such as polio eradication procedures and protection of health service supply chains in Libya hinged on individual relationships and the good will they engendered. However, high staff turnover--endemic to both security and health responders--threatened those working procedures. As one participant in the Haiti case study noted, “Turnover with NGOs is a huge problem…it causes a psychic shutdown. They think you are not cooperating but you’re shut down because you are having to start over yet again.” Global polio eradication workers in Afghanistan also noted this difficulty. “Frequent turnover among military staff can make it harder for the humanitarian community to establish strong working relationships and coordination mechanisms, but the often cited hurdle can be overcome if both parties are determined to make such relationships work” [26].

The creation of “problem solving spaces” was also important. Forums were needed where security and health system actors could meet, exchange information and ideas, provide feedback and address problems in an open and collaborative way. In Haiti, the Joint Operations and Tasking Center (JOTC) successfully created a mechanism to allocate security force resources to helping health service organizations. This mechanism afforded crisis response leadership an opportunity to organize and prioritize military assistance to health service groups [24]. The most successful example may be NATO’s “docking station” concept instituted during the Libya campaign, run by Joint Task Force Unified Protector in its operational command center. This task force established an office to serve as a clear point of contact with NGOs providing relief. The office went beyond public relations by providing a repository and response command for outside groups’ concerns. Critically, the office leadership was able to inform and influence NATO’s operational planning process based on the information it received from outside groups. It also had the authority to provide valuable information to these groups in return [50]. According to case study participants, this space to collate information and address shared concerns was influential in minimizing harm to indigenous health organizations in Libya [27]. Of note, the World Health Organization’s Health Cluster, which focused on humanitarian health action, rarely provided a productive forum for security actors to interact with health sector actors in our cases. The reasons for this were multiple including the fact that the Health Cluster is dedicated, by design, to humanitarian health action rather than indigenous health system protection. Discussions regarding how indigenous health organizations can regain functional status or how the sector will be financed were rare in health cluster meetings. Also, because humanitarian health guidelines stipulate minimal involvement of security actors in humanitarian health aid, the reception of security actors in the cluster meetings was mixed and many participants we spoke with felt it was inappropriate.

### Overarching policy issues to ISFs supporting health systems

Each case illustrated the presence of overarching policy barriers that confronted security organizations’ ability to best protect health systems. There were three core policy barriers:

### Lack of mandate

Security organizations’ mandates were most often focused on securing peace and targeted towards a specific group or source of conflict. The mandate to support health system actors and protect health system assets was often lacking or submerged implicitly in their scope of activities. Missing, in part, was the acknowledgement that functioning health systems can contribute to security and civilian protection.

Even when mandates were broad enough to include health system support and security organizations’ actions had a direct impact on the health system, in practice security organizations often did not view this within their organization’s role. For example, in Kosovo, lax budgeting and auditing procedures established and maintained with foreign donor support were tolerated for years. The weak procedures facilitated corruption in the health system. A Rule of Law mission was eventually fielded by the European Union but it had limited powers. Unable to help develop functioning, accountable systems, the mission was only able to take a reactionary role, charging Ministry of Health leadership with procurement fraud [25].

In Haiti, militaries providing tertiary care to civilians operated to provide short-term relief only. They were not mandated or empowered to support indigenous health system organizations, which would have not only provided services, but both restored and expanded capacity. Tertiary care services were withdrawn before indigenous care organizations had regained pre-earthquake functioning [24]. Militaries and peacekeepers in Haiti possessed assets that could have, if called upon in a timely way, blunted the cholera outbreak, including engineering capabilities, supply chain logistics, and surveillance tools. However political leadership seemed to view responding to public health emergencies—even ones with ramifications on the political stability of the country—outside of security forces’ mandates. Resources deployed were thus not exploited to fully strengthen the weakened health system [24,45].

### Lack of trust

Perceived differences in operations and missions nurtured distrust between ISFs and agencies supporting the health system. In fragile states, security organizations are not neutral; this is true even with a mandate to protect civilians and restore stability. Additionally, provision of health care is also not impartial; who gets access to health resources and when can favor one political or ethnic group over another. Health leaders responsible for overseeing the health system may also be aligned with one political group or another. Despite these complexities, non-governmental and many bilateral health agencies supporting the health system still strive to avoid exacerbating inequalities or supporting a single identifiable group. Working in collaboration with security groups or being seen to “cooperate” with them can politicize health care provision and may stoke conflict rather than lessen it. This conflict complicates aid agencies’ and other health focused organizations’ willingness to develop relationships and collaborations with security forces and organizations.

The cases demonstrate this tension, particularly in Afghanistan where militaries undertook direct health delivery activities in order to achieve numerous objectives that had very little to do with promoting civilian health or improving health system functioning. While initially perceived as an essential strategy by the International Security Forces Afghanistan, the practice was controversial and polarizing. It was later scrapped for its unanticipated harm to local and alliance relations and its paucity of useful data [43].

Security organizations also were hesitant to trust health stakeholders, as evidenced by their reluctance to share public health information. While there was tension around classified or possibly sensitive information, the practice of withholding or classifying information was also acknowledged to be largely reflexive. Security organization members were used to automatically protecting information. “Even when we were given an order to share information, we found it difficult because our systems are not set up to share”, one case participant acknowledged.

A second source of distrust understandably arose from the chaos of many agencies and actors working as part of the crisis response in the health system space. Health systems in the cases, like in many fragile and conflict-affected states, were flooded with a variety of actors and agencies jockeying for influence. The lack of a clear, leading, international agency responsible for organizing the efforts and for liaising with the Ministry of Health perpetuated poor communication, duplication of work, and gaps in health services. Security actors because of their prominent role in crisis response were often barraged by a wide array of NGO workers, politicians, and agency representatives requesting partnership or a strategy change. Yet, who to trust, with whom to share information or from whom to solicit opinion was not clear.

### Lack of institutional interoperability

In all four cases, security organizations’ involvement in the health system was ad hoc and subsequently, institutional interoperability with health groups was lacking. A stark example was in Haiti when the US military was tasked with reopening the airport and reconstituting supply routes throughout the country. The technical and logistics capability displayed by the US military was impressive. Yet, the lack of communications procedures, policies around sharing information, and prioritizing supplies blunted their effectiveness. Health workers, for example, struggled to find medications because the crush of supplies, often unlabeled, coming through fragile supply lines managed by the US military took weeks to organize and distribute. This contributed to a spike in mortality two weeks after the earthquake because wounded civilians were unable to access adequate medical resources [33].

However difficult the logistics were, the underlying difficulty was that forums for stakeholders to collaborate and improve distribution effectiveness in a timely way simply did not exist until weeks after the earthquake [24]. This was true throughout the cases. Despite the fact that ISFs were involved in health system logistics to some extent, as in Haiti, interoperability in terms of common understanding, joint problem solving space, communication platforms, and patient tracking mechanisms, didn’t exist.

## Discussion

This analysis shows that ISF involvement in health go beyond interactions with humanitarian health service providers in fragile states. Health sector governance, indigenous health services, and health information systems were the three health system ‘building blocks’ documented frequently in the cases.

In some instances ISF engagement was critical to health system preservation, as when military forces reconstituted medical supply chains in Haiti. In others, their impact was dangerous and destructive, as in the delegitimizing of the Ministry of Health in Afghanistan during counterinsurgency operations, or with the unintended introduction of, and slow response to, the cholera epidemic in Haiti. Moreover, the cases uncovered multiple opportunities wherein ISFs could significantly improve aspects of health sector preservation and functioning. The potential to better protect health workers in Kosovo and care for war-wounded in Libya, or leverage their epidemiologic and surveillance systems to detect and respond to public health threats in Kosovo, stand as prime examples.

Since ISF health sector engagement can present unprecedented opportunities for health system protection and recovery, as well as pose dangers and risks, it should follow that there exist mechanisms by which health sector actors can work with ISFs to carefully manage their engagement.

Yet, to date this is not the case. Rather, interactions between security and health sector actors are best characterized as ad hoc, frequently disjointed efforts that evolve and congeal long after opportunities to protect health sectors and avoid unintended consequences has passed. The case studies suggest several parameters for the types of communication and consultative mechanisms necessary to manage ISF involvement in supporting health sectors in CAFS. Namely, these mechanisms need to be able to address a wide array of problems, be nimble enough to accommodate rapidly changing circumstances, leverage the power of personal relationships, and be able to address the tensions that can arise between security and health system supporting strategies.

With these parameters in mind, we suggest two approaches to improving ISF-health sector interactions: building expertise within ISFs with respect to health system protection and recovery, and developing a network of high level liaisons across security and heath sector supporting organizations involved in fragile states.

### Health security teams

Knowledge of health systems within ISFs is necessary to avoid unintended negative impacts on health systems and to be able to recognize and exploit opportunities to use ISF assets to support health systems. Some security policymakers may feel uncomfortable developing this expertise within ISFs because it seems so far afield from traditional security training. Indeed, few security organizations appear to invest in programs or foster career paths devoted to developing experts in public health emergency response much less health system strengthening. However, at least within militaries, there is long precedence of public health and medical advising. This experience has, until recently, focused primarily on keeping troops healthy and addressing public health threats to personnel, rather than the public health toll of conflict on civilians. The rubrics of ‘stability’, ‘peace building’ and ‘civilian protection’ missions have the potential to change this focus because these concepts emphasize supporting effective indigenous governing institutions to varying degrees [51,52]. ISFs could thus build health system support into their long-established health training programs with the reassurance that this would be in line with increasing their capacity to conduct these ‘non-traditional’ types of security missions.

While deployment of this expertise could take many forms, we recommend ‘Health Security Teams’ that would be deployed on the ground. These teams would differ from traditional civilian-military or NGO liaisons because they would have as a primary goal health system support and would have specific training in public health and institution building in fragile states. Further, there would be clarity that their jobs are not to overshadow health sector actors or supplant health sector support with military objectives but rather to manage the security organizations role in health system support initiatives. Health-Security Teams’ work would be two-fold.

The first would be to address and optimize the myriad aspects of health system building blocks impacted by ISFs in a particular fragile setting. The cases offer a blueprint, or ‘building-block’ based checklist of issues for health–security teams to consider. Regarding supporting health sector governance, for example, health-security teams might approach health system leaders to plan how separate health systems for security personnel or investments in the emergency and disaster preparedness systems can dovetail with Ministry of Health initiatives and avoid competition between different fragile state ministries for health workers, public health emergency assets, or donor funding. Health security teams part of ISF Rule of Law missions might seek out opportunities to build transparent and accountable budgetary, auditing, and health system performance information systems so as to improve transparency and stave off corruption. In periods of acute crisis, health security teams could generate proposals for how the ISF could stem the loss of health sector instruments of governance like health records and epidemiologic surveillance systems. The full checklist of potential issues for health-security teams to consider, broken down by building block, is included in Table 1. Table 1 is organized by three levels of crisis acuity facing the health sector. In ‘periods of quiescence’ threats to the health system are minimal or insidious. Opportunities for strengthening health system functions exist. During acute crises, significant losses of health sector assets and degradation of health system functions are possible. Protection of assets and mitigating loss of health system functions is the priority. In post-crisis periods, efforts to restore health system assets and functions are made, or significant reforms to health system building blocks are undertaken.

**Table 1:** **Possible health system support activities for international security forces by building block**

**Building block**	**Crisis acuity**		
	**Periods of quiescence**	**Acute crisis**	**Post-crisis**
Governance	Develop baseline knowledge of health system leaders, providers, and financing mechanisms	Assess opportunities to save instruments of health governance such as medical and vaccination records, payroll systems, procurement data, health communication and information systems.	Consider how medical systems for indigenous security personnel and disaster preparedness systems relate to the civilian health sector. Work with health sector leadership to develop approaches to these health sub-systems that will avoid competition with the civilian health sector for health workers, public health emergency assets, and donor funding.
		Consider offering communications and logistics support to health sector leadership if it is necessary to help them manage public health crises	
			If the ISF is engaged in anti-corruption initiatives, support or advocate for transparent health sector procurement and budgetary processes
Health information	Develop baseline knowledge of epidemiologic surveillance, census, and health performance systems	Assess if the ISF’s epidemiologic, environmental health surveillance, and public threat detection systems can feed into or strengthen civilian surveillance systems.	Assure robust protocols are in place regarding how public health threats will be monitored, who will be notified of their presence, and what collection of organizations and actors will act to mitigate them.
	Contribute to or advocate for donors to fund baseline health data collection systems in fragile states		
		Assess if ISF situational awareness tools can yield information about the status of health sector assets, and if these tools can contribute to the overall international health system support effort.	
Indigenous health service delivery	Consider opportunities to strengthen operational ties with leading health system institutions so these ties will be in place in case of acute civilian health crisis	Assess opportunities to protect health sector infrastructure. Be mindful that many instances the best protection strategy may be one of ‘passive support’ where maximum distance is kept between ISFs and indigenous health organizations and institutions. Likewise develop and implement plans for how ISFs can offer maximum protection to health sector workers	Consider if and how ISF logistics and engineering assets can bolster public health infrastructure or mitigate public health threats
		If ISFs are going to provide direct health care to civilians, this care should be done in the context of supporting health system organizations regain pre-crisis operational levels.	

The potential health supporting activities listed in Table 1 are broken down by the health sector building blocks the cases revealed were most often affected by international security forces (ISFs). It is also organized by acuity of crises facing the health sector. In ‘periods of quiescence’ threats to the health system are minimal or insidious. Opportunities for strengthening health system functions exist. During acute crises, significant losses of health sector assets and degradation of health system functions are possible. Protection of assets and mitigating loss of health system functions is the priority. In post-crisis periods, efforts to restore health system assets and functions are made, or significant reforms to health system building blocks are undertaken.

Throughout this project we have distinguished between ISF involvement in supporting health sectors in fragile settings and participating in humanitarian health aid delivery. The considerations presented in this table are not meant to replace careful analysis of how ISF actions will impact humanitarian organizations delivering health aid or on humanitarian space generally. The Interagency Standing Committee and Global Health Cluster guidelines provide an excellent tool for assessing the risks to humanitarian principles presented by ISF involvement in humanitarian health operations, for example [20]. In some instances, as when ISFs decide to provide health care directly to populations, both an analysis of how this will impact the indigenous health sector mentioned above, and an analysis of how it will impact humanitarian space needs to be undertaken.

The second function of Health Security Teams would be to calibrate the involvement of an ISF’s engagement in health systems to the political climate. As the cases demonstrate, the goals of ISFs as well as civilians’ perception of ISF involvement can vary widely, depending of the context, and change overtime. In Afghanistan overt and visible signs of ISF engagement in health elevated distrust of the population and did appear to politicize the health sector. In Kosovo, the opposite was true at least in the context of 2012 when health sector leaders solicited coalition force involvement in health sector issues because of their perceived fair mindedness. OCHA describes different types of interactions between humanitarian and military actors in conflict zones, ranging from minimal interaction to collaboration [17]. A similar range of interactions may be necessary between ISF members and health sector actors. Judgments about how and when to adopt different types of inter-organization relationships can only be made by those familiar with the context, stakeholders, and issues over time. ISFs need to come to the field prepared to make these judgments with counterparts in health system supporting organizations.

### Policy networks

Developing field level expertise alone within ISFs is insufficient; they alone would be unable to address the overarching policy concerns that cut across multiple fragile state contexts.

To meet this additional requirement, we recommend development of a network of liaisons across major health sector and security organizations. The past two decades have seen the emergence of networks of practitioners to tackle a range of thorny foreign policy problems including cross-border law enforcement, economic trade issues and environmental policy problems [53]. Networks of liaisons, across humanitarian and UN agencies, such as the Interagency Standing Committee have been particularly influential in generating policies that impact military-humanitarian relationships.

While both security and health sector organizations presently field a diverse group of liaisons on occasion, the network we propose would be more formalized and participants would share several unique features. They would be knowledgeable about their own organization, yet share a common training in supporting institutions, particularly health, in fragile states. Importantly, they would wield enough leverage in their own organizations to influence its operations. They would have significant field experience and ongoing contact with field level health sector expertise. They would participate in forums designed to foster the growth of working personal relationships and mutual understanding.

Members of this network of practitioners would ideally hail from health sector supporting organizations such as the World Bank, health sector donor agencies and their implementing partners, the Global Fund for AIDS, Tuberculosis and Malaria, the Global Alliance for Vaccines and the World Health Organization, in conjunction with major ISFs including NATO, the African Union, the European Union, and major bilateral militaries often engaged in fragile states through peacekeeping missions.

The network could address at least three key policy issues: how issues of information sharing between security and health sector actors will be managed in fragile settings; best practices with respect to bridging civilian health sectors, indigenous civilian emergency response systems and indigenous security forces’ health services; and how security organization mandates can best incorporate health system protection.

Issues of information sharing include deciding how data about public health threats will be collected, shared and acted upon across health system and security actors; when health information will be classified and released, and to whom it will be shared; and what assurances security organizations can offer that health information will not be used inappropriately.

The network of liaisons could create joint guidance as to how to best reconcile parallel health systems. In several of the cases security organizations funded and built health systems for indigenous security forces or civilian emergency response systems that sat outside of the Ministry of Health. These health ‘subsystems’ sometimes created problems in terms of health sector funding and human resource shortages. Better coordination among the sub-systems is needed for long term sustainability of the health resources and personnel.

With respect to mandates, this network could advise policy makers on how to incorporate health system protection into security organization mandates. This mandate should not indicate that security organizations be in charge of health systems, but rather that they should view protecting and supporting health systems as essential part of the overall objective of achieving security in the long term.

During conflict or crisis, security members of this network would already have working relationships with key health sector support organizations that could facilitate rapid problem solving. Using NATO’s Libya response as a model, these security network members could open and run “docking stations” whereby health sector actors and even non-network members could quickly engage the security organization to exchange information and address emerging problems. Further these security network members could draw on the expertise and information of the health-security teams to help design operations to minimize harm to health sectors and realize opportunities to salvage threatened health sector assets like health workers, infrastructure and information systems.

Of particular importance, this network of liaisons could help manage the tensions that inevitably arise between security strategies and health sector strengthening efforts. There are skeptics within the global health community wary of security organizations because of the fear that their involvement will lead to subjugation of health to military objectives. The use of health projects in counterinsurgency campaigns has crystalized this skepticism. It is instructive to note, however, that these campaigns came about in the absence of established consultation and communication forums between health and security actors, not because of them. In the absence of standing forums, it took non-governmental organizations (NGOs) and security analysts in Afghanistan ten years of protest and confrontation to convince coalition forces to draft and adopt doctrines that discouraged the use of direct health provision projects as counterinsurgency tools, for example.

The Afghanistan experience strongly suggests that health sectors supporters need a voice within ISFs to define and advocate for health-friendly ISF strategies. This is not to say health system support and security strategies will never be in conflict, or that health sector support will or should always win out when they do conflict. As the Overseas Development Institute’s report, “State building for peace” points out, there is often tension between efforts to build equitable and high functioning institutions in fragile states (state building) and brokering the compromises among warring factions and foreign interests contesting control over state institutions (peace building) [54]. Those interested in building health institutions may often be in tension with those attempting to improve security and foster peace. Yet, these tensions make it all the more important that there are consultative forums and open lines of communication among professionals if these tensions are to be managed transparently, conscientiously, in a timely way and to the benefit of all involved.

### Limitations

This study possesses several inherent limitations. With respect to the cases, data and conclusions were drawn from impressions, memories and opinions of individuals from four qualitative case studies conducted year(s) after an event, perhaps reporting partial or inaccurate recollections of events.

A further limitation derived from the make up of the investigation team. On one hand having a combined team of civilian academics and military analysts facilitated outreach to a wide variety of stakeholders and key informants. We did not receive any overt refusals to participate. On the other hand, the sensitivities some participants may have had about speaking with NATO-affiliated analysts or Harvard researchers may have made them more or less reluctant to offer criticism or share concerns. Further, not all interviews were conducted in person in the field. Subtle but important information gleaned from face to face interviews and field visits may have been lost. Finally, this was a qualitative project and subject to the tacit subjectivity of the research team.

With respect to the cross case analysis, all comparative methodological approaches used to study social phenomena must be considered with caution. The presence of phenomena across cases may be spurious, rather than due to the reasons proposed by the analyst and the definition of the phenomena itself may be questionable since societal or organizational behaviors are open to the interpretation of the framers of the research. With respect to this study, the findings are necessarily broad since the outcome variable of interest—the health sector building blocks—are themselves general, and sometimes overlapping themes, rather than discrete and well defined variables. The findings, grouped in terms of these broad themes, need to be unpacked through future narrow investigations. Our hope is that this study will provide the rational, evidence-based foundation to that will allow future researchers to formulate this focused research.

## Conclusions

Our case studies illustrated the complex context and challenges to health systems in crisis-affected fragile states. In most cases, these challenges pre-existed the crisis and will last for years afterwards, contributing to persistently weak health systems and subsequent high morbidity and mortality. The cases demonstrated both opportunities and risks in international security organizations responding to help protect health systems and support their recovery and reconstruction. Governance, health information systems and health services are the most accessible points of impact and there is opportunity pre-, during, and post- crisis to better engage ISFs all three areas.

This cross-case analysis and included recommendations are intended to provide a platform and a start of a continuing discussion of security organizations’ role and responsibilities. For example, the recommendation of establishing a permanent network of liaisons across major health sector and security organizations will require time, coordination and agreed commitment on the part participants that health system preservation and support in CAFS in desirable and possible. Open forums of discussion and consultation will be essential but will also require joint commitment and broad representation to be effective and worthwhile. This study is intended to trigger the needed analysis and discussions to facilitate supportive mandates and policies among both ISFs and other health stakeholders in these settings.

## Abbreviations

EULEX: European Union Rule of Law Mission in Kosovo
CAFS: Conflict-AFFECTED OR FRAGILE STATES
GPEC: Global Polio Eradication Campaign
ISAF: International Security Assistance Force
ISF: International Security Forces
JOTC: Joint Operations and Tasking Center
KFOR: North Atlantic Treaty Organization’s Kosovo Forces
KNIPH: Kosovo National Institute of Public Health
MINUSTAH: United Nations Stabilization Mission in Haiti
MKSF: Ministry for the Kosovo Security Force
NATO: North Atlantic Treaty Organization
NGO: Non-governmental Organization
OCHA: Office for Coordination of Humanitarian Affairs
PAHO: Pan American Health Organization
UN: United Nations
UNMIK: United Nations Mission in Kosovo
UNAMA: United Nations Assistance Mission in Afghanistan
US: United States of America
USAID: United States Agency of International Development
WHO: World Health Organization
